# Challenges and shifting paradigms in clinical trials in oncology: the case for immunological and targeted therapies

**DOI:** 10.3332/ecancer.2019.936

**Published:** 2019-07-05

**Authors:** Diana Carolina Sotelo-Rodríguez, Alejandro Ruíz-Patiño, Luisa Ricaurte, Oscar Arrieta, Zyanya Lucia Zatarain-Barrón, Andrés F Cardona

**Affiliations:** 1 Foundation for Clinical and Applied Cancer Research—FICMAC, Bogotá 100110, Colombia; 2 Clinical and Translational Oncology Group, Institute of Oncology, Clínica del Country, Bogotá 100110, Colombia; 3 Thoracic Oncology Unit and Laboratory of Personalized Medicine, Instituto Nacional de Cancerología (INCan), México City 14080, Mexico

**Keywords:** master protocols, umbrella design, basket design, immunotherapy cancer, clinical trials

## Abstract

The advent of immunotherapy has undoubtedly changed the current standard for cancer treatment. Immunotherapy offers the possibility of achieving excellent results—a new alternative for patients with advanced-stage or relapsed disease. Nowadays, the progress made in tumour biology has led to multiple advances in clinical and translational cancer research. Many oncogenic pathways responsible for tumour growth and metastases have been described and, consequently, multiple new cancer therapeutic agents have been developed and are under current investigation. Due to this rapid increase in knowledge and pharmaceutical development, traditional clinical trials designs have encountered major limitations. The pharmacological differences (in toxicity profiles and effectiveness patterns) between immunotherapy and chemotherapy have caused traditional clinical trials to evolve in order to meet this emerging need. This review focuses on the different options pertaining to clinical trial design that have arisen in the field of immuno-oncology, as well as the challenges of accurately interpreting traditional survival analyses within this novel area of cancer medicine.

## Introduction

Cancer research is a field that has consistently grown over time; the increase in knowledge has led to the development of multiple new target-specific therapeutic agents. Immunotherapy is a remarkable example. In 1891, William B. Coley inoculated *Streptococcus pyogenes* and *Serratia marcenses* in a patient with inoperable sarcoma and noted that the immunologic response generated destruction of the tumour cells in that patient. This was the first evidence supporting the widely suspected anti-tumour role played by the human immune system [1]. The observations by Dr. Coley lay the groundwork for several transcendent discoveries in the field of immunology, including the advent of immune-checkpoint inhibitors [2]. In 2011, the Food and Drug Administration (FDA) granted approval for the first immunotherapeutic drug: ipilimumab (Bristol Myers Squibb), for use in patients with advanced melanoma [3]. Today, the field of immuno-oncology continues to grow, with more impetus than ever, with the rise of several agents which have proven to be effective in the treatment of multiple malignancies. In addition to their efficacy, immunotherapy agents have also gained advantage due to their favourable toxicity profile, with less severe adverse effects due to their specific immunologic anti-tumour nature, compared to traditional cytotoxic schemes.

Unfortunately, and almost inevitably, these advances have caused significant increases in the cost of cancer care. Adams *et al*. estimated that the total cost of the process of research and development of new cancer drugs, including preclinical and clinical testing, is approximately $1 billion dollars. In addition, the capitalised preclinical, clinical, and total costs per new drug have exponentially increased over time, and although this increase is reflected in all the areas of healthcare, it is particularly magnified in the area of cancer medicine, where recent reports estimate a cost between $648 million–$2.7 billion USD [4, 5]. A possible explanation for the exceedingly high cost of bringing cancer drugs to the market, compared to other disease-type drugs, is in part due to the level of complexity and length of time required to conduct phase III cancer clinical trials [6]. These increasing costs could have led to a change in clinical trial design that looks for optimisation of indication and conduction. In the specific case of immunotherapy, a change of direction in the design of clinical trials was adamantly needed.

This is because for the first part, this group of therapeutic agents is very diverse; it includes checkpoint inhibitors, monoclonal antibodies, cancer vaccines and adoptive T-cell therapies [7]. Moreover, not all the agents have been shown to have efficacy across all the tumours and to complicate the matter even more, tumour heterogeneity drives different responses in patient subgroups, conditioning the degree of clinical benefit observed in each individual patient. Another important consideration is temporality, while clinical benefit in patients receiving standard chemotherapy is generally observed during active treatment, this is not true for immunotherapy, where both effectiveness and toxicity are delayed, sometimes several months, after treatment [8]. Adding all these factors to high costs of development and approval, the need to design clinical trials that optimise indications, taking into consideration the mechanism of response, its variation according to genomic alterations and the specific adverse effects observed with these novel drugs was warranted.

## Types of biomarker-based designs

The design of clinical trials has been modified due to the advent of targeted therapy. Biomarkers play an important role in immunotherapy since they identify patients who are more likely to reap higher benefit from a particular therapy [9]. This approach renders biomarkers the basis for clinical trials with molecularly guided recruitment.

### Enrichment or “targeted”

**Figure 1.**
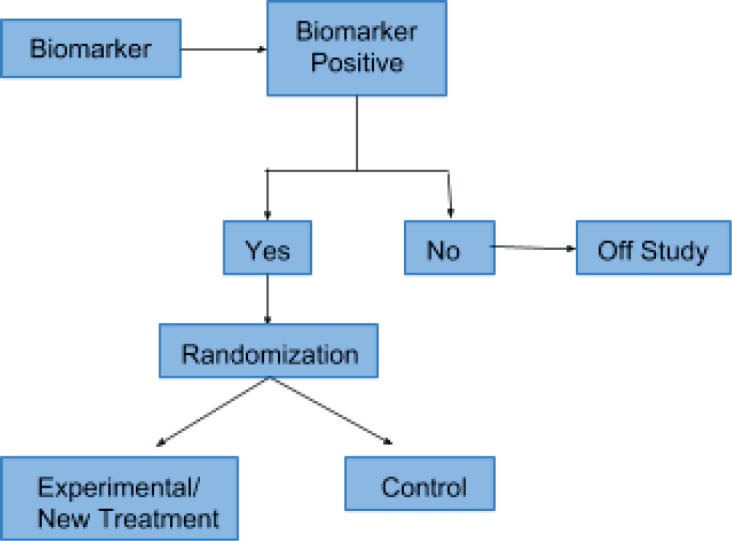
Enrichment or targeted trial design.

This type of design was first described by Simon and Maitournam [10]. Initially, only patients who are positive for a particular biomarker are recruited for the study. Thereafter, the population is randomised into experimental and control groups. This type of clinical trial is the perfect framework for the evaluation of treatment efficacy in a biomarker-positive subpopulation. Simon *et al*. [11] describe that this design is appropriate for phase II studies and it has been implemented in trials evaluating drugs for *BRAF*-mutated melanoma (vemurafenib) and *ALK*-positive lung cancer (crizotinib). Figure 1 shows the enrichment or targeted trial design.

### Marker by treatment interaction

Plays an important role in the exclusion of patients according to their biomarker status, each subpopulation is randomised to the experimental versus control treatment group.

### Modified marker strategy

Used in diseases with one or more approved therapies. The objective is to identify marker subpopulation with the most benefit to a specific therapy [12].

## Master protocols

Master protocols are next-generation clinical trial designs that evaluate the combination of several molecular markers and their targeted therapies. These protocols rose from the current need for innovative trials that allow simultaneous assessment of multiple treatments in one disease or one treatment in multiple diseases. It is one single protocol that addresses many questions with faster results and at a lower cost. The tumours from patients enrolled in these types of trials are analysed with next-generation sequencing and/or immunohistochemistry. The final purpose is to collect large amounts of data. Based on the results, patients are distributed to sub-studies [13]. The main advantage of these master protocols is the efficient patient population selection [14]. There are three different designs included within the master protocols. Even though these designs vary greatly between one and other, they also share many features. For example, they require more planning efforts and, in that way, obtain high-quality data and increased trial efficiencies. Because of this, these novel designs of clinical trials can last longer but provide precise results [15].

**Figure 2.**
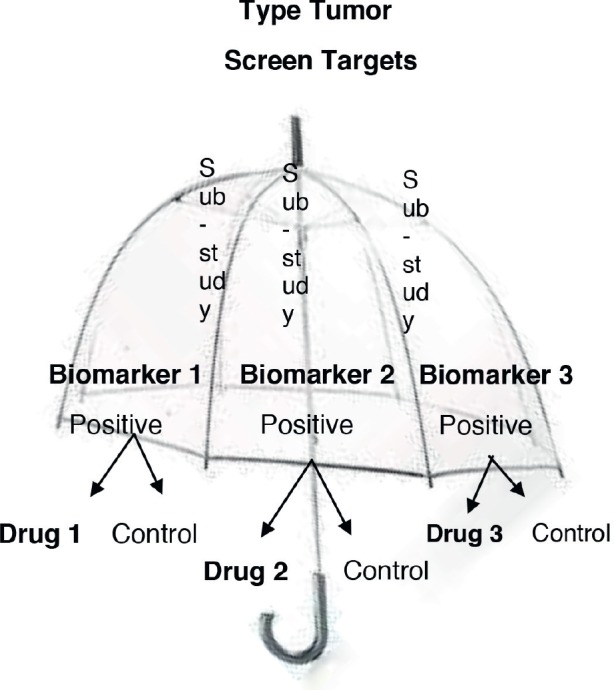
Umbrella trial design.

**Figure 3.**
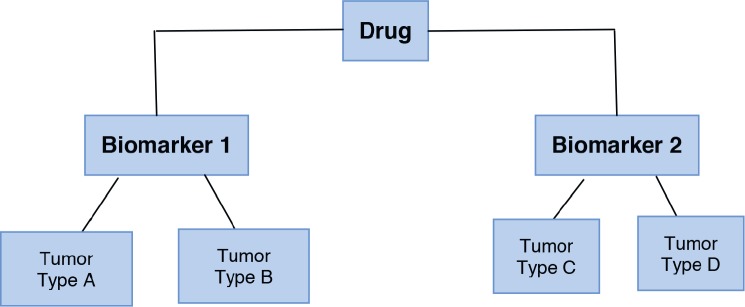
Basket trial design.

These master protocols include the following designs:

1. ***Umbrella design:*** Patients with the same disease are recruited, later the different genetic alterations are identified, and different drugs are given to them according to their molecular characteristics. All the targeted agents are investigated “under the umbrella of one disease.” This design is time and cost-effective [15]. Also, these trials include multiple treatments and multiple biomarkers in the same study allowing randomised comparisons. An example from this design is “*The Adjuvant Lung Cancer Enrichment Marker Identification and Sequencing—ALCHEMIST*.” The aim of this study was to identify patients with early-stage lung cancer with *EGFR* and *ALK* mutations and to evaluate drug treatments targeted against these molecular alterations [16]. Figure 2 shows the umbrella design.
2. ***Basket design:*** Patients with the same genetic mutation are included regardless of the type of cancer they have. These trials are based upon the principle that drug effectiveness is dependent on their target and not on the tumour type. They are useful to study a single targeted therapy in the context of multiple diseases or disease subtypes. It allows the separate analysis of patients with different tumour types and identifies the effect of the drug on all the patients as one single group [15, 17]. Furthermore, it can help in the development and study of specific biomarkers in rare tumours. For example, the study of “*Vemurafenib in multiple non-melanoma cancers with BRAF V600 mutations”* enrolled patients with BRAF V600 mutation-positive cancers that were not melanoma or papillary thyroid carcinoma [18]. One important limitation of basket designed-studies is the fact that in some cases the histological tumour type can be a better predictor of treatment response than the biomarkers themselves [19]. Figure 3 shows the basket trial design.
3. ***Platform design:*** These studies may be designed as umbrella or basket trials but with the difference that these studies permit the inclusion or exclusion of new therapies or patient populations along the trial development. In other words, this type of study runs perpetually and changes the medication evaluated or patients. The idea is to mature treatment strategies for a pathology across populations or medications. Analyses, which per definition are interim analyses, determine the efficacy of an intervention and, therefore, lead to the inclusion or exclusion or molecules or patients [14].

## Survival analysis in immunotherapy

Survival analysis is one of the main statistical tools used in oncology nowadays. It is used to determine differences between treatments and/or interventions. It mathematically demonstrates an existing difference among two or more treatment groups as of the occurrence of an expected event, such as death or progression, in determined follow-up time. This, in turn, is called time-to-event analysis.

In patient follow-up, there are some issues that can potentially cause many traditional statistical tools to lose validity. First, the absolute follow-up of subjects is very difficult to assure. Either in a cohort or an intervention group, an expected but unknown quantity of patients will be lost to follow-up. This dropout can occur at any moment and can potentially lead to incorrect conclusions. The statistical paradigm in these cases could be either to include them in the analysis and assume dropout as an event, increasing the odds of encountering type 1 error; or exclude them in the follow-up, and therefore lose power and risk getting a type 2 error. A middle ground for both perspectives is usually undertaken. This approach is called *censoring* and takes the patients into account for analysis until the dropout time is achieved and, after that moment, they are excluded. Since this dropout occurs after study initiation, it’s correctly called *right censoring* [20]. Additionally, since the dropout is expected to occur randomly, it should not be related to prognosis. This is a core principle behind the Kaplan–Meier estimator (KM-Estimator). Proposed by Edward L. Kaplan and Paul Meier in 1958, it is one of the most widely used methodologies in survival analysis. Briefly, it calculates the probability of presenting the event given the number of individuals at risk at a defined moment. It also adjusts for censored observations. Results of this estimator are plotted in a survival graph in which each observation of event is presented in a ladder curve [21].

Another aspect to have in mind is the behaviour of the event presentation at follow-up. Normally, when modelling time-to-event data, survival functions, or the functions that model the probability of event occurrence in a determined time frame, should be known. Since the conduction of cohort or clinical study is designed to establish the survival behaviour of a set of patients, these functions, *a priori*, are therefore unknown. To compensate for this uncertainty, an assumption on the occurrence of events is made. This is known as the *proportional hazard rate assumption*. The idea behind this concept is to model occurrence as exponential functions (i.e., *exponential model*) in both groups. This relates to the effect in hazards as constant in time for each individual group through follow-up [22]. This assumption becomes crucial, especially in the study design given that it is required to express the effect size of intervention for sample size estimation and the interpretation of the KM-estimator [23].

Finally, in order to objectively define differences between survival curves and treatment groups the *log-rank test* is traditionally employed. It is based on the same assumptions as the KM-estimator, and therefore both are analysed together in the study. It behaves similarly to an *X*^2^ test. The principle lies in establishing a difference between expected and observed values on the times of the events. The sum of these individual differences equals the value of the statistic on an *X*^2^ distribution. After assigning a *p* value with regard to the degrees of freedom, the null hypothesis can, or cannot, be rejected [24].

Since the introduction of immunotherapy into oncology in recent years, results and the representation of survival curves and their corresponding statistical considerations have had some problems with the previously mentioned methodology. Ipilimumab, the first monoclonal antibody to be proven to be effective in unresectable/metastatic melanoma revealed fascinating results in terms of survival behaviour. After 10 years of follow-up, a pooled analysis of 1861 patients yielded a median overall survival (OS) of 11.4 months. Interestingly, after achieving a plateau at around 3 years, around 22% of patients remained alive for the rest of the follow-up. Additionally, the behaviour of the ipilimumab-treated patients was similar to chemotherapy-exposed ones in the first 4 months, with a separation of the curves following that period [25].

These findings showed a drastic difference compared to the traditional chemotherapy trials. Moreover, the behaviour of the curves demonstrated that the proportional hazards rate assumption is not met. This is the case since in the initial months, hazards rates remain proportional, but as time progresses, hazard for the event also diminishes. Additionally, other results of immunotherapy trials have even shown an early crossing of the curves, complicating matters even more [26]. These phenomena have become known as either *long term survivors, delayed treatment effect and functional cure****.***

**Figure 4.**
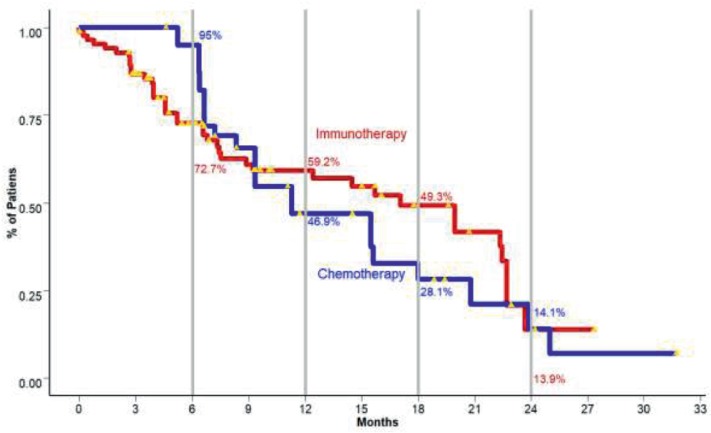
In the presented image, two cohorts of patients with metastatic non-small cell lung cancer, one treated with standard chemotherapy and the second with immunotherapy and their correspondent % of overall survivors are presented. Each step along the staircase-like curve represents events, in this case, deaths. The yellow triangles are the censored observations. Curve intersection is observed at around 9 months (Disclaimer: This figure is original of the authors and was submitted for publication as part of an original article in another journal. No copyright transference has been made at this point).

These concepts are better explained with Figure 4.

Further issues arise when designing future immune-oncology studies. Since the main differences are going to be seen in the long-term survivor’s group, an adequate number of patients should be randomised in order to guarantee a significant difference in this aspect, increasing the size of the sample. Additionally, the implementation of a model different from the aforementioned exponential should be warranted. One possible solution for this latter aspect is the use of a better-fitted model: the *Weibull*. This model has several advantages, including the fact that it represents hazards as a function of time, correcting for the disproportionality of hazard rates. Furthermore, it also offers the inclusion of covariates and describes long-tailed distributions, representative of long term survivors [27].

Moreover, estimation of a traditional endpoint such as OS or progression-free survival (PFS) also seems to be inadequate. Taking the previous example of metastatic melanoma, OS for ipilimumab and dacarbazine-treated patients reached a median of 11.2 months compared to 9.1 months in the dacarbazine monotherapy group [28]. When comparing median OS, the magnitude of effect doesn’t seem dramatic, since the true effect is observed in long-term survivors. Furthermore, the log-rank test becomes inadequate for assessing statistical differences, since this test gives each observation an equal weight in the final statistic calculation, thus, in turn, cutting short the effect on long term survivors. A possible alternative is the usage of *weighted log-rank tests*. These tests put weights of importance on observations based on their moment of occurrence during follow-up, correcting for the loss in statistical power. The *Fleming–Harrington test* was proposed as an alternative. As a core characteristic, it possesses two parameters that can be tuned to match specific weight allocation on the survival curves. Inherently, a problem presents when giving a priori values to these parameters since the point of curve divergence cannot be estimated prior to the conduction of the study [27]. All in all, these problems, and specifically, the underestimation of the magnitude of effect by traditional survival outcomes state the necessity for other endpoints different to OS and PFS per se.

*Milestone survival* defined as the cross-sectional evaluation of survival at predefined moments is an alternative. Evaluating the percentage of patients alive at two years, in the case of melanoma and ipilimumab, should in theory be representative of long-term survivors. Key aspects for this issue rely on defining the milestones at the study’s design, conduction of follow-up to the milestone, and finally, preventing if possible interim analysis, since the desired effects will present later, and therefore be missed [22, 27]. Limitations to this approach include the effects of censored observations as well as its inability to describe the behaviour of the survival curves [29]. Additionally, since there is no consensus on the specific points in time to be set as milestones, this lies on the investigator´s preference. Normally, such endpoints are set for OS and PFS at 6, 9, 12, or 24 months. As a way to offer robustness in the analysis, milestones should be indicated before the conduction of the study in the clinical trial statistical analysis plan.

## Conclusions

In summary, immunotherapy has not only introduced interesting concepts both in terms of biological effects and therapeutic changes but also in statistics and clinical trial design, conduction and interpretation. Although not ideal, milestone survival might be considered as a valid strategy to demonstrate differences in survival. Further implementation of other statistical methods and consensus are still required to reach a final conclusion on definitive methods.

## Conflicts of interest

Dr Arrieta reports personal fees from Pfizer, grants and personal fees from Astra Zeneca, grants and personal fees from Boehringer Ingelheim, personal fees from Lilly, personal fees from Merck, personal fees from Bristol Myers Squibb, grants and personal fees from Roche, outside the submitted work. Dr Cardona reports grants from Merck Sharp & Dohme, Boehringer Ingelheim, Roche, Bristol-Myers Squibb and The Foundation for Clinical and Applied Cancer Research—FICMAC., other from Pfizer, Boehringer Ingelheim, Astra Zeneca, MSD, BMS, Celldex, Roche, personal fees from Merck Sharp & Dohme, Boehringer Ingelheim, Roche, Bristol-Myers Squibb, Pfizer, Novartis, Celldex Therapeutics, Foundation Medicine, Eli Lilly and Foundation for Clinical and Applied Cancer Research—FICMAC, outside the submitted work. All other authors have no competing interests to declare.

## Funding

This study did not require funding.
